# Natural bamboo powder and coffee ground as low-cost green adsorbents for the removal of rhodamine B and their recycling performance

**DOI:** 10.1038/s41598-023-48354-4

**Published:** 2023-12-06

**Authors:** Thi Sinh Vo, Muhammad Mohsin Hossain, Kyunghoon Kim

**Affiliations:** School of Mechanical Engineering, Sungkyunkwan University, Suwon, 16419 Korea

**Keywords:** Environmental sciences, Environmental chemistry, Environmental impact

## Abstract

Bamboo and coffee, which are abundant and inexpensive, have been used as green adsorbents for the adsorption of industrial dye rhodamine B (RB). Bamboo and coffee are natural sources of cellulose, hemicellulose, and lignin, making them promising green materials for industrial dye removal. The effects of various adsorption conditions, such as contact time, temperature, dose of bamboo powder (BP), coffee ground (CG), initial concentration of RB, and pH values of RB solution, were measured. Consequently, the kinetics of RB adsorption onto bamboo and coffee was in accordance with the pseudo-second-order model, with an activation energy of 29.51 kJ mol^−1^ for bamboo and 27.46 kJ mol^−1^ for coffee. The Langmuir model is well fitted to the whole adsorption period at different temperatures, in which the increase in the tested temperature has improved the adsorption capacity (*i.e.,* BP: 6.76 mg g^−1^/30 °C, 6.96 mg g^−1^/40 °C, 7.64 mg g^−1^/50 °C and CG: 6.53 mg g^−1^/30 °C, 6.80 mg g^−1^/40 °C, 7.51 mg g^−1^/50 °C). Moreover, the spontaneous nature of the adsorption was based on the negative Gibbs free energy values obtained (*i.e.,* from − 11.09 to − 14.30 kJ mol^−1^ [BP] and from − 10.34 to − 13.07 kJ mol^−1^ [CG]). These revealed that RB adsorption occurred at physical and chemical adsorption states. In addition, the recycling capability of adsorbents was determined in five cycles. Therefore, these materials are promising candidates for low-cost adsorbents.

## Introduction

At present, dye pollution is regarded as a major issue in various applications, *i.e.,* dyeing, leather, textiles, plastics, and food industries, etc.; hence, these colored matters dramatically impact to environmental problems. The use of different organic dyes in these industries are toxic and induce drawbacks to the water source. Of these, rhodamine B (RB) is known as a cationic dye, which is often employed as a staining fluorescent dye in biology and biotechnology applications^1^. RB is also considered as a well-known hydrophilic xanthene dye in the paper printing, leather, paint, textile, coloured glass and plastic industries; besides, this dye is often combined with herbicides to reveal where they have been used. As such, the available existence of these complex aromatic rings can show more stability in the molecular structure of organic dye inducing to being unfavorble to biodegrade; thus, requirements in the organic dye removal with a cost-effective approach is considered to be a big challenge for researchers. Conventional water purification methods, such as physicochemical and biological treatments, are almost ineffective for dye removal because of dye stability^2–4^, while adsorption is an effective technique in organic dye-contaminated water/wastewater treatment to release the dye-related colors from the water/wastewater, majorly thanks to flexibility, simple design, low cost, and easy operation^5–8^. Consequently, many researchers have focused on developing adsorbent materials to efficiently remove dyes from wastewater^9–12^. Previous studies have successfully predicted the adsorption isotherms of dyes on various materials such as magnetic Fe_3_O_4_-modified papaya seed^13^ and guava leaves^14^ powders, surface-modified lychee peels^15^, activated carbon^16^, activated carbon from oil palm wood^17^, peach stones^18^, phoenix tree leaf powder^19^, coir pith carbon^20^, sepiolite^21^, and pumice powder^22^. Among these, activated carbon is a well-known material with a high surface area (~ 3000 m^2^ g^−1^), and it is known for its excellent adsorption ability for all kinds of pollutants^23^. However, the high cost associated with activated carbon regeneration limits its practical applications. Therefore, the use of low-cost adsorbents made from bio-materials, natural materials, and waste materials from agriculture and industry has become increasingly popular as a cost-effective alternative for water treatment^24,25^.

**Figure 1:**
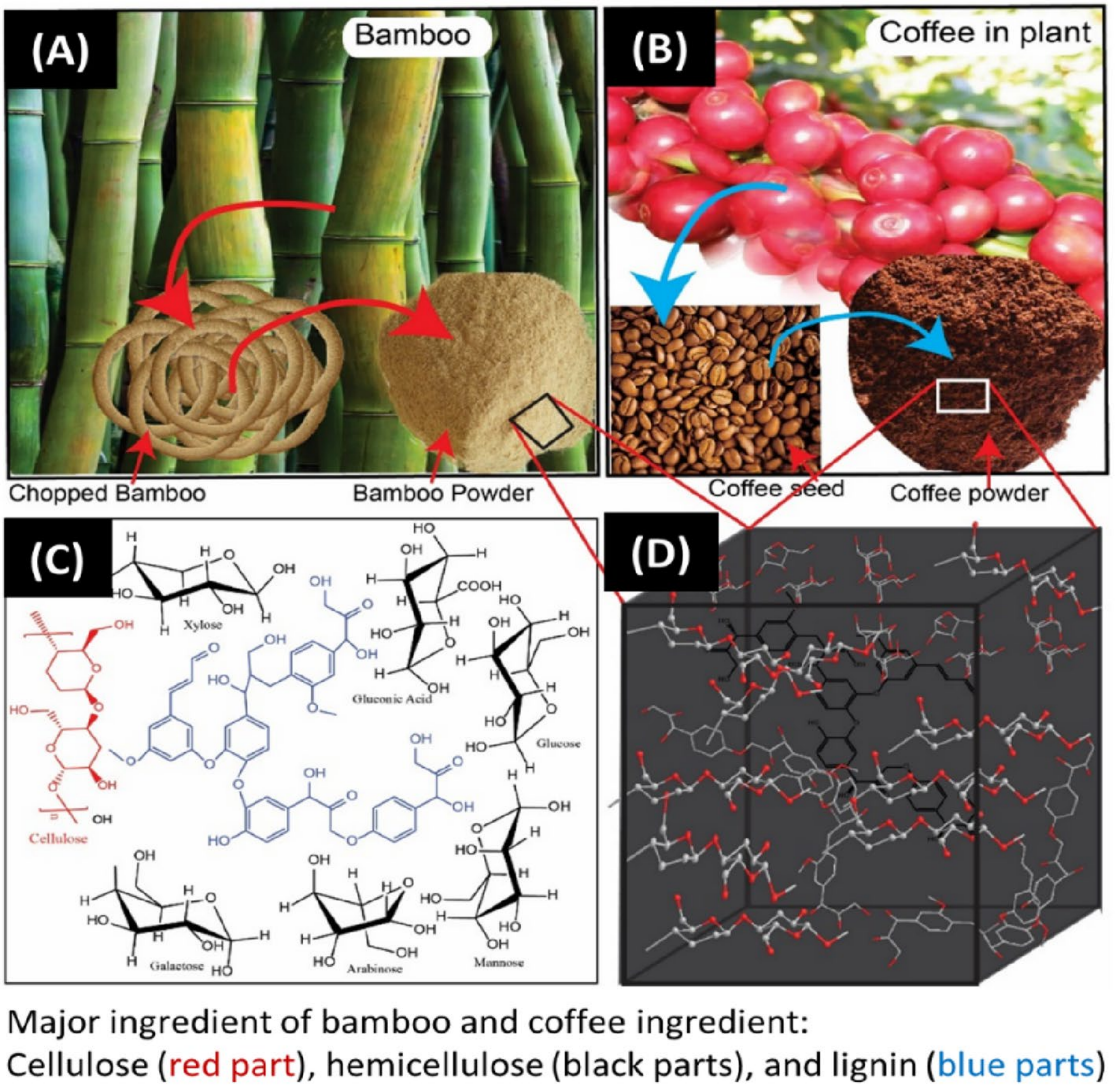
Natural bamboo and its powder form prepared from its chopped form (**A**), natural coffee in plants and its ground form prepared from its seed form (**B**), major ingredients of BP and CG (**C**), and 3D form of the available chemical ingredients contained in BP and CG (**D**).

Recently, a wider range of low-cost adsorbents that can effectively remove dyes, organics, and heavy metals from water in an environmentally-friendly manner have been developed. One promising new resource that has been identified is non-chemically modified bamboo powder (BP) and coffee ground (CG) because of its cost-effectiveness, renewable nature, and environmental friendliness^26–28^. In particular, BP (Fig. 1A) is a natural composite material that is abundant in tropical countries, which is also considered as a composite material because of the cellulosic fibers implanted in a lignin matrix^29^. For CG (Fig. 1B), it consists of polysaccharides, hemicelluloses, lignin, pectin, cellulose, and a small amount of extractives^30^. The surface of CG contains active functional groups (amino, hydroxyl, and carboxyl groups; Fig. 1C,D); thus, CG has great application potential in dye removal because of its cost-effective, renewable, and environmentally friendly features^31^. In addition, the negatively charge carboxyl group is considered as the major functional group in the adsorption of cationic dye molecules, but it is not effective in the adsorption of anionic dye molecules^32,33^.

Herein, BP and CG are used directly with non-chemical modification for adsorption purposes. Although the adsorption capacity of these non-chemically modified adsorbents is limited, their cost is nearly zero, which are washed only with distilled water, dried at room temperature, and directly applied in the adsorption process. To our knowledges, the dye adsorption performance using these non-chemical-modified green adsorbents has rarely been investigated, and the adsorption mechanism has not been adequately interpreted, at same time that the adsorption capacity has not still been compared among the non-chemically modified BP and CG. Notably, the cost of these low-cost green materials was almost zero, because they are only cleaned by distilled-water and air-dried, and then were directly used for RB adsorption process. Therefore, this study aims to examine the adsorption capacity of RB (*i.e.,* a cationic dye that is widely used as a colorant and a tracer fluorescent with toxic and carcinogenic nature) by using the non-chemically modified adsorbents (BP and CG), in which this investigation may be the first until now. The effects of contact time, temperature, the dose of non-chemically modified adsorbents, initial RB concentration, and pH values of RB solution are also investigated in detail. Moreover, experimental data are analyzed through the adsorption equation of kinetics, isotherms, and thermodynamics; the specific parameters for each model and their recycling ability are also investigated. Therefore, these materials can be considered as potential low-cost adsorbents.

## Materials and methods

### Materials

BP and CG were provided from Hanyang Advanced Materials (South Korea) and Trung Nguyen Coffee (Vietnam), respectively (Fig. 1A,B). They were washed several times with distilled (DI) water under magnetic stirring to remove residual organics, dirt, and color. Then, the residues of CG were used with a powder size of 140–280 μm using sieves with various mesh sizes (CHUNG GYE SANG GONG SA, KOREA). RB was obtained from Sigma Aldrich. Filter papers (*Φ* = 47 mm) were purchased from CHMLAB GROUP. DI water was utilized through a Milli-Q ultrapure water purification system. All methods were carried out in accordance with relevant guidelines.

### RB adsorption experiments

**Figure 2:**
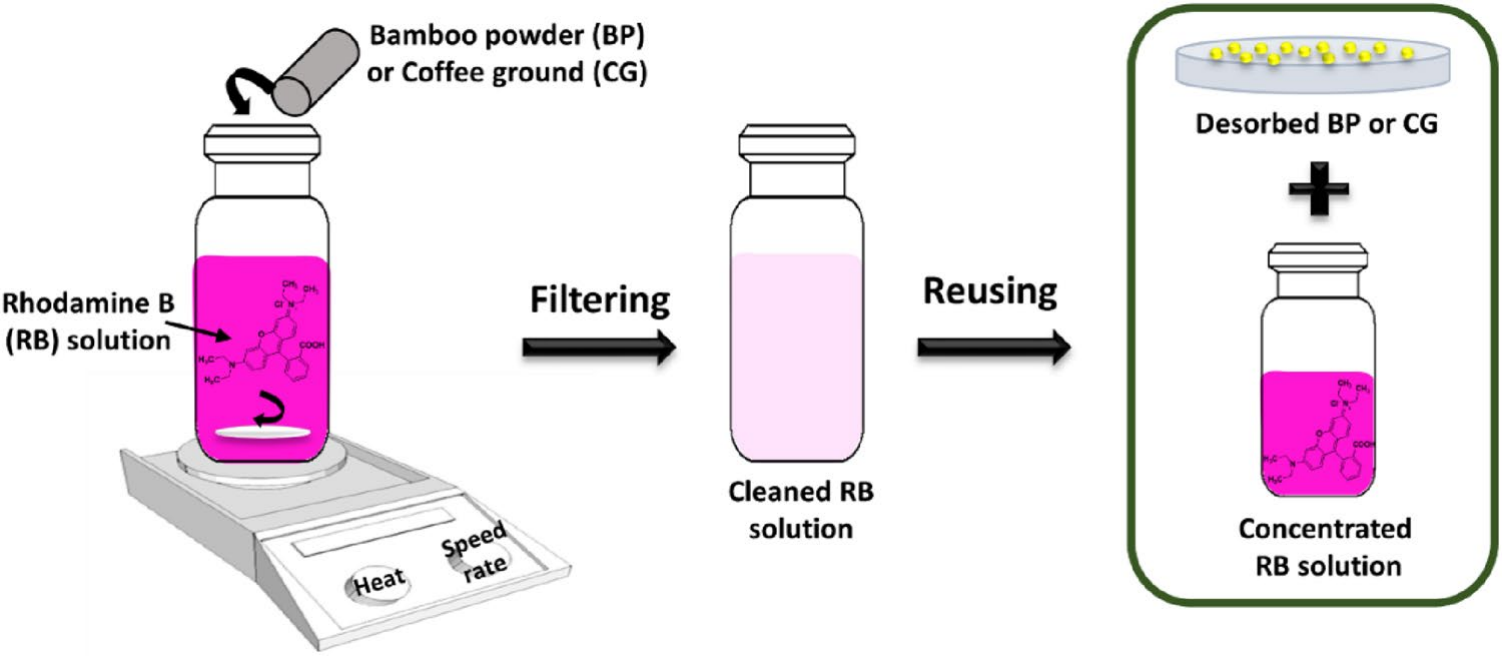
RB adsorption using the non-chemically modified adsorbents.

Adsorption experiments were conducted by stirring (200 rpm) the non-chemically modified adsorbents (BP and CG) into 50 mL of RB solutions for 230 min to reach equilibrium at various temperatures (30 °C, 40 °C, and 50 °C; Fig. 2). The effect of adsorbent dose (0.05, 0.1, 0.3, 0.5, and 1.0 g) was studied in 50 mL of 20 mg L^−1^ RB solutions at various temperatures. Next, initial RB concentrations (5, 10, 20, 30, and 50 mg L^−1^) were also utilized to examine their effect at the above-mentioned temperatures during adsorption. In addition, the effect of pH values (2, 3, 6, 8, and 9) was investigated in 6 g L^−1^ adsorbent dose with 20 mg L^−1^ of RB at the above-mentioned temperatures. Finally, RB concentrations in aqueous solutions after filtering using a filter paper were determined on the basis of the absorbance value at 550 nm through UV–vis analysis. Moreover, RB solutions of C_o_ = 20 mg L^−1^ were utilized in the reuse process to investigate the recycling performance of non-chemically modified adsorbents (Fig. 2). The non-chemically modified adsorbents after adsorbing RB were washed several times with ethanol, and the regenerated sample was used to adsorb RB. The RB removal efficiency (%) at time t was calculated using Eq. (1); C_o_ and C_t_ (mg L^−1^) correspond to RB concentrations at an initial time and time t (the tested period is 0–300 min).

1 $$\mathrm{Removal }\left(\mathrm{\%}\right)=\frac{({\mathrm{C}}_{\mathrm{o}}-{\mathrm{C}}_{\mathrm{t}})}{{\mathrm{C}}_{\mathrm{o}}} \times 100$$

#### Adsorption kinetics

Kinetic studies were performed at various constant temperatures by stirring 0.3 g of the non-chemically modified adsorbents with 50 mL of 20 mg L^−1^ RB solutions in each glass vial (200 rpm). All aqueous samples of each glass vial were filtered by using filter papers to remove solid materials at various time intervals of 0–300 min. Then, RB concentrations in aqueous solutions were examined on the basis of the absorbance value at 550 nm through UV–vis analysis. The amount of adsorption at time t (q_t_, mg g^−1^) was calculated by using Eq. (2); C_o_ and C_t_ (mg L^−1^) correspond to RB concentrations at an initial time and any time t; V (L) and m (g) refer to the volume of the RB solution and the mass of non-chemically modified adsorbents utilized, respectively.

2 $${\mathrm{q}}_{\mathrm{t}}= \frac{{(\mathrm{C}}_{\mathrm{o}}-{\mathrm{C}}_{\mathrm{t}})\times \mathrm{V}}{\mathrm{m}}$$

#### Adsorption isotherms

Adsorption isotherms were conducted using 0.3 g of non-chemically modified adsorbents in 50 mL of different initial RB concentrations (5–200 mg L^−1^), and each sample was stirred for 230 min (30 °C, 40 °C, or 50 °C) to obtain an equilibrium mixture (200 rpm). Then, the final RB concentration in the aqueous solution was analyzed at ~ 230 min. The amount of adsorption at equilibrium (q_e_, mg g^−1^) was calculated using Eq. (3); C_o_ and C_e_ (mg⋅L^−1^) correspond to RB concentrations at initial and equilibrium times; V (L) and m (g) refer to the volume of the RB solution and the mass of non-chemically modified adsorbents utilized, respectively.

3 $${\mathrm{q}}_{\mathrm{e}}= \frac{{(\mathrm{C}}_{\mathrm{o}}-{\mathrm{C}}_{\mathrm{e}})\times \mathrm{V}}{\mathrm{m}}$$

### Analysis instruments

The structure of materials was measured by using a FESEM JSM-7600F instrument. FT-IR spectroscopy was determined in a wavenumber range of 4000–400 cm^−1^ using an FT-IR spectrophotometer (IFS-66/S, TENSORR27, Bruker Co.) and the KBr method. X-ray diffraction (XRD) was conducted using an X-ray diffractometer (D8-ADVANCE, Bruker Co.) in the *2θ* range of 5°–70°. Thermal gravimetric (TG) curves were achieved in a temperature range of 0–800 °C with a heating rate of 20 °C min^−1^ using a Seiko Exstar6100 instrument. UV–visible spectroscopy was measured at room temperature using a SpectraMaxM5 instrument.

## Results and discussion

### Characterization of the non-chemically modified adsorbents

**Figure 3:**
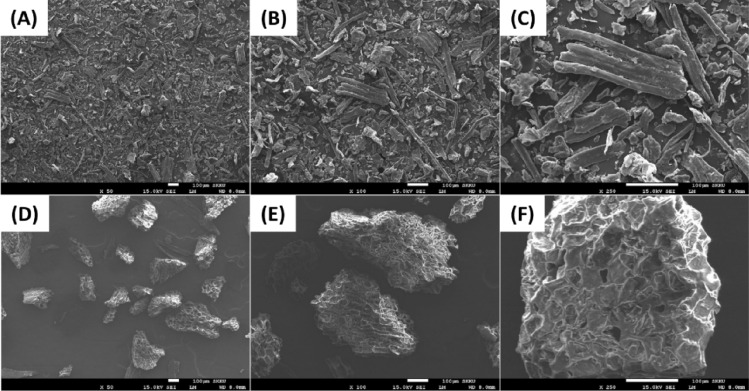
SEM images of BP (**A–C**) and CG (**D–F**) with 50 × (**A, D**), 100 × (**B, E**), and 250 × (**C, F**) magnifications.

For a adsorbent, its physical morphology is able to be an important factor to evaluate removal efficiency of organic dyes from water/wastewater. Figure 3 shows the SEM images of non-chemically modified adsorbents (BP and CG) before adsorption. In particular, BP is observed as fibers with a smooth surface and a size range of 1–30 μm (Fig. 3A–C). By contrast, CG is observed as porous grains, with a rough surface (Fig. 3D–F), a size range of 140–280 μm, and various cavities. These cavities could be characterized as channels onto the surface of CG instead of pores. The enlarged image of non-chemically modified adsorbents is shown in Fig. 3C,F), which suggests the hierarchical morphology of BP and CG. As shown in Fig. 3, the non-chemically modified adsorbents are micrometer in size, and they consist of numerous nanomaterials that are hundreds of nanometers in size. Therefore, the non-chemically modified adsorbents with different morphologies and sizes affect the dye removal ability from aqueous solution and the collision between them and the adsorbate.

**Figure 4:**
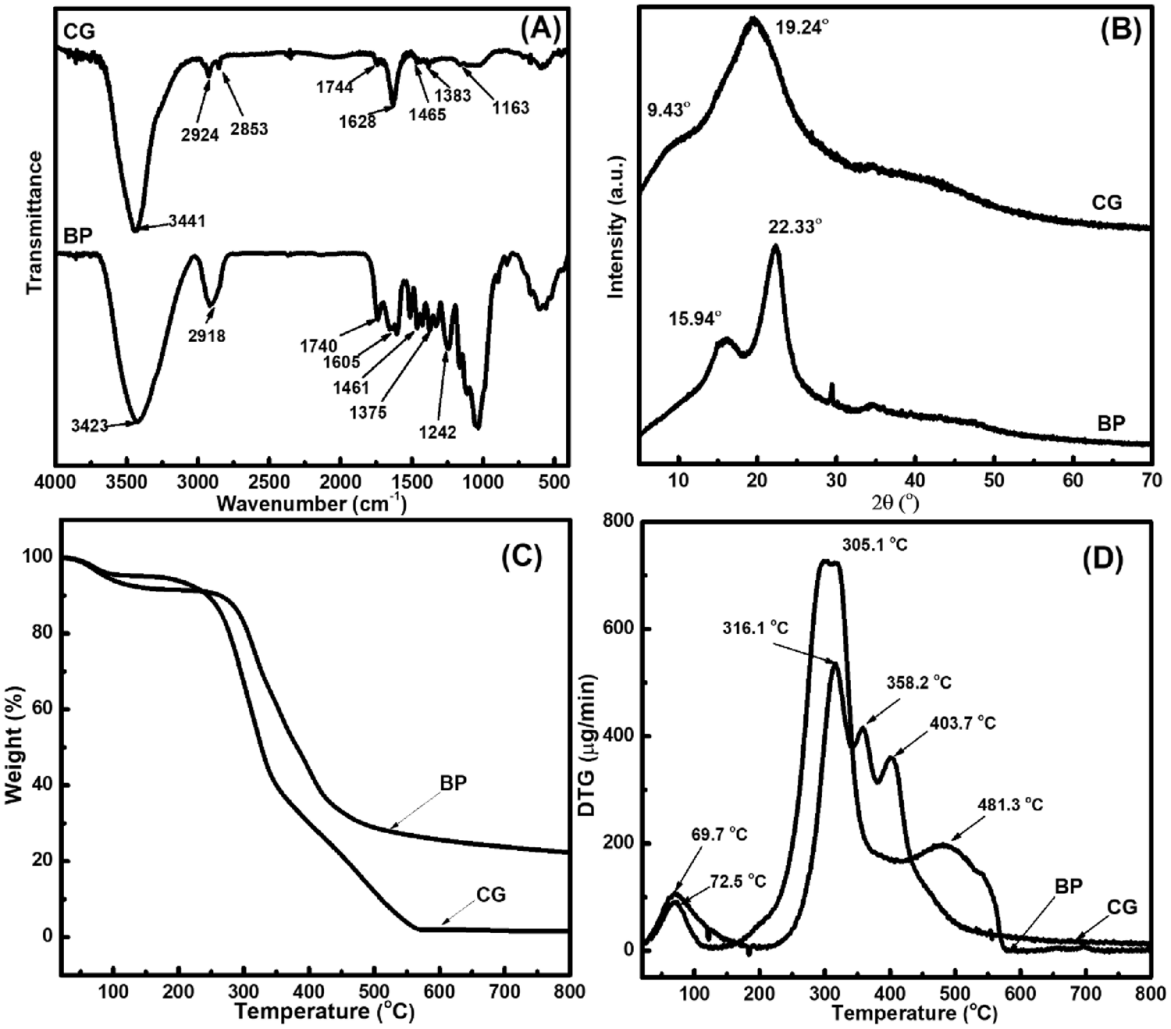
FTIR (**A**) and XRD (**B**) spectra as well as TG (**C**) and DTG (**D**) curves of the non-chemically modified adsorbents.

Aside from the morphological characteristic of the adsorbents, their chemical and crystalline features were also analyzed via FTIR spectroscopy and XRD pattern. The FTIR spectrum of non-chemically modified adsorbents was measured to determine the functional groups of BP and CG. As shown in Fig. 4A, BP and CG spectra show lignocellulosic characteristics based on the major constituents (*i.e.,* fatty acids, lignin, hemicellulose, cellulose, and polysaccharides)^34^. In particular, the broad peak at 3423 and 3441 cm^−1^ belongs to the O–H stretching vibration in the BP and CG spectra, respectively. The peaks at 2924 cm^−1^ (asymmetry) and 2853 cm^−1^ (symmetry) belong to the C–H stretching vibration in the CG spectra; however, only one peak of the C–H stretching vibration is found at 2918 cm^−1^ (asymmetry) in the BP spectrum. Moreover, the peaks at 1740–1605 cm^−1^ (BP) and 1744–1628 cm^−1^ (CG) correspond to the carbonyl C=O stretching of hemicellulose and chlorogenic acids^34,35^. The peaks at 1461–1375 cm^−1^ (BP) and 1465–1383 cm^−1^ (CG) are involved in the −CH_2_ and –CH_3_ bending modes (hemicellulose, lignin, etc.). The peaks at ~ 1242 and 1163 cm^−1^ correspond to the C–O–C bonds of chlorogenic acid, lignin in BP, and CG spectra. In addition, the broad peak at 1100–990 cm^−1^ belongs to the C–O–H bond of polysaccharides^36^ in the BP and CG spectra.

In the meantine, XRD analysis was performed to confirm the structure of non-chemically modified adsorbents (BP and CG, Fig. 4B). The diffraction pattern of such adsorbents exhibited two broad peaks at 15.94° and 22.33° (BP) as well as at 9.43° and 19.24° (CG), indicating that cellulosic materials appear in BP and CG^34^. Furthermore, hemicellulose and other components of the non-chemically modified adsorbents have an amorphous structure^34^. In addition, TG analysis was used to investigate the thermal properties of non-chemically modified adsorbents (Fig. 4C,D). In particular, the first stage of CG and BP occurs at 69.7 °C and 72.5 °C respectively, because of water evaporation and volatile compounds. The next stage (300 °C–400 °C) is related to the decomposition of polysaccharides and several fats in BP and CG; moreover, the decomposition temperature of hemicellulose and cellulose is observed at 305.1 °C (BP), 316.1 °C, and 358.2 °C (CG)^34,37^. The last stage (> 400 °C) could be assigned to the formation of carbonaceous materials and consolidation of carbon structures^34,37^.

### RB adsorption testing

Adsorption capacity is governed by several operational factors; herein, the effects of contact time, temperature, the dose of non-chemically modified adsorbents, initial RB concentration, and pH value of RB solution were investigated. In addition, the adsorption rate on smaller materials is faster than that on larger ones because of the large external surface area of smaller materials, which increases the collision between the adsorbate and adsorbent (*i.e.,* RB and the non-chemically modified adsorbents). Thus, the movement of small materials in solution is faster than that of large ones, as well as their shear is more on their surface^38,39^.

#### Effect of contact time and temperature

**Figure 5:**
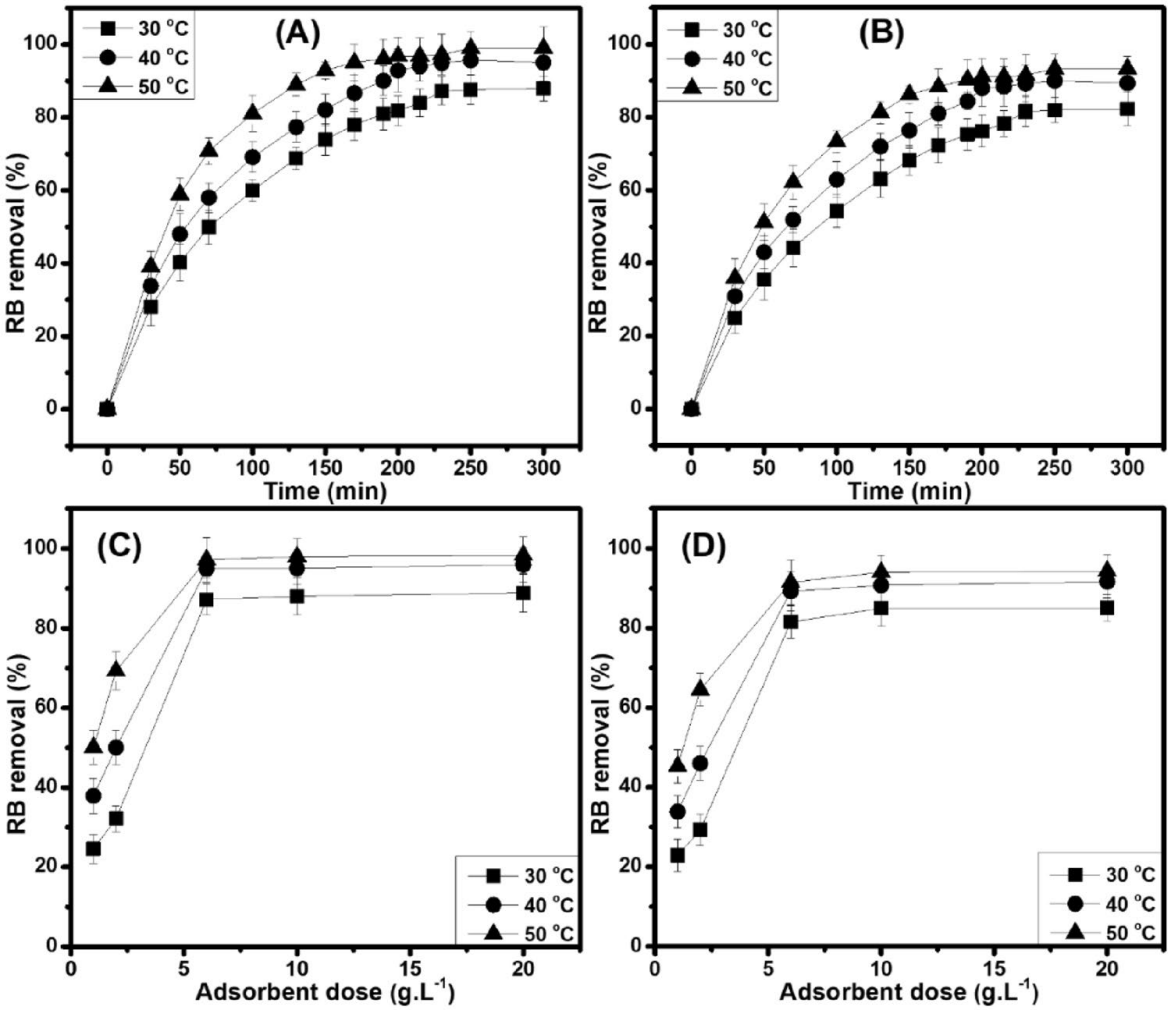
RB removal efficiency (C_o_ = 20 mg L^−1^, pH = 6) onto BP (**A**) and CG (**B**) (6 g L^−1^) at different time and temperatures. RB removal efficiency (C_o_ = 20 mg L^−1^, pH = 6) onto BP (**C**) and CG (**D**) at 230 min.

The effect of contact time and temperature is shown in Fig. 5A,B). The RB removal percentage increases quickly with contact time, which indicates a possibly strong interaction between RB and the non-chemically modified adsorbents (BP and CG). Here, the equilibrium time is selected at 230 min for 20 mg L^−1^ of RB concentration (30 °C–50 °C), and the RB removal percentage remains almost constant. In particular, the RB removal percentage at 230–300 min is 87.3–87.9% (BP) and 81.6–82.2% (GC); 94.9–95.0% (BP) and 89.2–89.3% (CG); and 97.2–98.9% (BP) and 91.5–93.3% (CG) at 30 °C, 40 °C, and 50 °C. Consequently, the contact time of 230 min is chosen to be an optimal time for next investigations of the adsorption process.

The contact time occurring between the non-chemically modified adsorbents (BP and CG) and the adsorbate (RB) is important to the adsorption process. In particular, the adsorbed adsorbate take a short contact time in the physical adsorption, which is contrary to the chemical adsorption because the chemical bonds between the adsorbent and adsorbate can reach equilibrium with a longer contact time. Furthermore, the uptake of adsorbate is quick at the initial time of the contact period (*i.e.,* 215 min/30 °C; 200 min/40 °C; 170 min/50 °C for BP and CG) because of a large number of available active sites on the surface of non-chemically modified adsorbents (BP and CG)^20^. Then, it slows down to obtain near equilibrium. In addition, the RB adsorption is considered as an endothermic reaction because of the increasing adsorption capacity with temperature. In promoting the diffusion rate of RB molecules crossing the external boundary layers and being into the internal pores of non-chemically modified adsorbents (BP and CG), the temperature should be increased to decrease the solution viscosity, and different temperatures will change the equilibrium capacity^21^.

#### Effect of the non-chemically modified adsorbents’ dose

The effect of adsorbent dose (1–20 g L^−1^) is also investigated using contact time, initial RB concentration, and pH value at different temperatures as constants **(**Fig. 5C,D). Consequently, the overall trend for different temperatures is the same, and the increasing adsorbent dose (1–6 g L^−1^) increases the RB removal percentage because of the increasing adsorbent (BP and CG) surface area and available adsorption sites. However, the RB removal percentage remains almost unchanged with the increase in adsorbent dose (6–20 g L^−1^), which can be due to the overlap or aggregation of the surface area of non-chemically modified adsorbents (BP and CG) to RB, thereby increasing the diffusion path length ^40^. Hence, the adsorbent dose of 6 g L^−1^ is used as an optimal parameter for next investigations of the adsorption process. Comparing between BP and CG, the RB removal percentage onto BP is higher than that onto CG at different temperatures (*i.e.,* BP: 24.6–88.9%/30 °C, 37.9–96.0%/40 °C, 50.1–98.3%/50 °C and CG: 22.9–85.1%/30 °C, 33.9–91.7%/40 °C, 45.3–94.3%/50 °C with an increase in the adsorbent dose [1–20 g L^−1^]), which is primarily due to the morphology and size of BP. Based on the SEM result, BP is a fiber with a smaller size compared with CG. As previously described, the collision between RB and the non-chemically modified adsorbents with a smaller size and larger external surface area increases; thus, the movement in the solution is faster than that of large ones^38,39^.

#### Effect of initial RB concentrations

**Figure 6:**
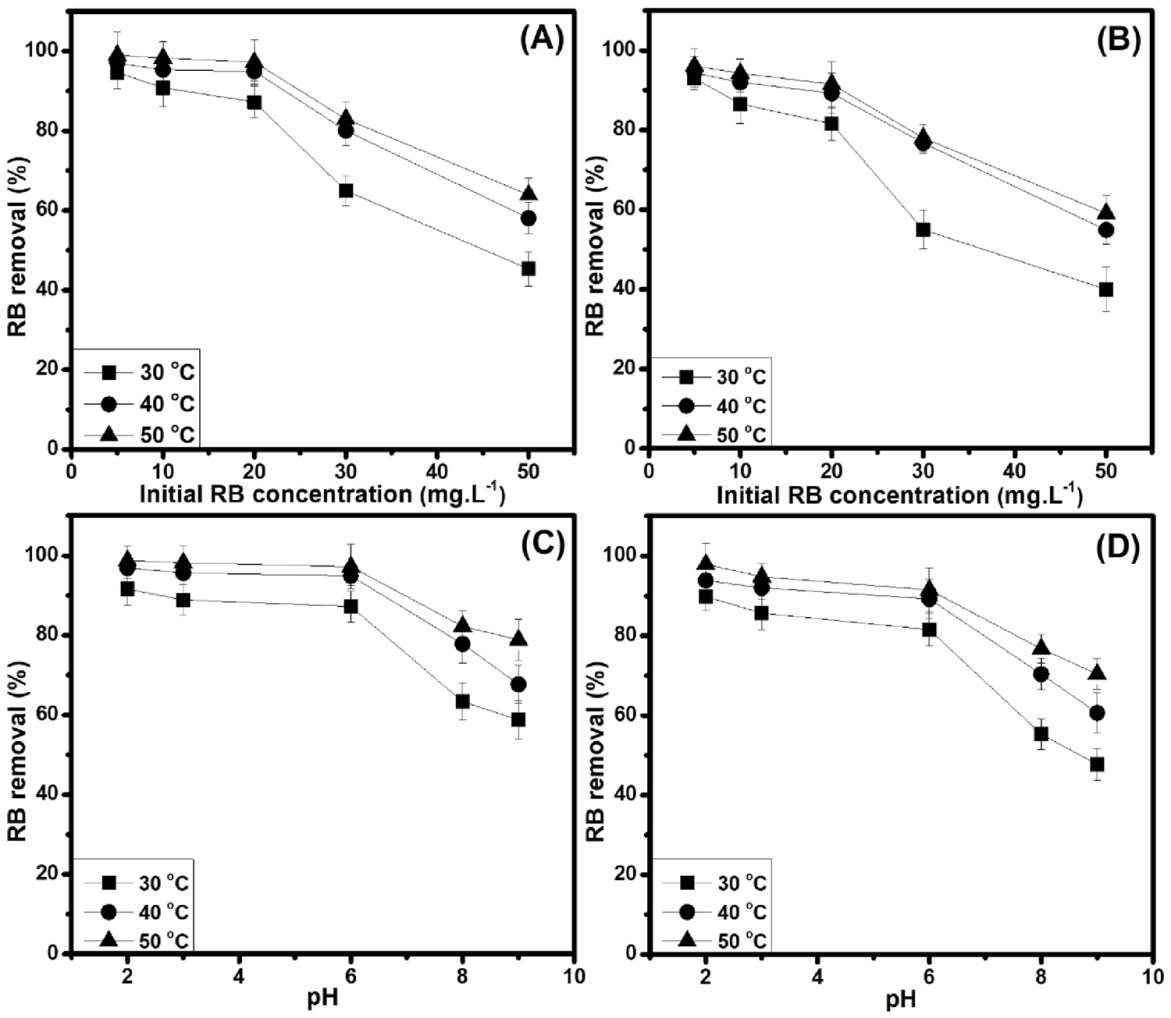
RB removal efficiency onto BP (**A**) and CG (**B**) (6 g L^−1^) with different initial RB concentrations at 230 min. RB removal efficiency (C_o_ = 20 mg L^−1^) onto BP (**C**) and CG (**D**) (0.3 g) with different pH values at 230 min.

Initial RB concentrations (5–50 mg⋅L^−1^) are used to examine the effect of RB at different temperatures with the adsorbent dose, contact time, and pH value as constants (Fig. 6A,B). Consequently, the RB removal percentage decreases with the increase of initial RB concentrations (*i.e.,* BP: 94.6–45.3%/30 °C, 97.1–58.1%/40 °C, 99.0–63.9%/50 °C and CG: 93.0–40.0%/30 °C, 94.3–54.9%/40 °C, 96.1–59.1%/50 °C with an initial RB concentration of 5–50 mg L^−1^) because of possible chemical and physical interactions between the adsorbate (RB) and the non-chemically modified adsorbents (BP and CG). This result is similar to the overall trend for different temperatures. In particular, the RB removal percentage rapidly decreases in RB concentration of 20–50 mg L^−1^ because of the overlap of the adsorption sites and the lack of adsorbent^40^. Thus, the RB concentration of 20 mg L^–1^ is used to conduct next investigations for the adsorption process. More specifically, the RB molecules can be also blocked the physical traps through a thin blocking layer formed rapidly on the adsorbents’ top-surface position at a higher RB concentration, so the RB molecules’ entering probability inside the other physical traps is less. Also, this decrease in RB removal efficiency probably involves to the used active sites’ amount on the adsorbents limited at the higher RB concentrations. By contrast, the thin blocking layer can be formed slower or negligible at a lower RB concentration, and consequently, the RB molecules can enter and be sealed inside the other physical positions. From these, the RB removal efficiency onto these low-cost green adsorbents reduces at the higher RB concentrations, and it has an opposable trend at the lower RB ones.

#### Effect of pH value

The pH value of a corresponding solution is considered as an important condition in the whole adsorption process, which affects the dye chemistry and the surface charge of non-chemically modified adsorbents in the aqueous solution^16,21,22,41,42^. In the RB adsorption removal, the effect of pH value (2 − 9) is presented in Fig. 6C,D, which is similar to the overall trend for different temperatures. The results indicate that the RB removal efficiency decreases with the increase of solution pH (*i.e.,* BP: 91.6–58.8%/30 °C, 96.9–67.7%/40 °C, 98.8–78.9%/50 °C and CG: 89.9–47.7%/30 °C, 93.9–60.6%/40 °C, 98.0–70.4%/50 °C with a RB solution pH range of 2–9]. In particular, the RB removal percentage decreases rapidly in the solution pH range of 6–9 (*i.e.,* BP: 28.5%/30 °C, 27.3%/40 °C, 18.3%/50 °C and CG: 33.8/30 °C, 28.6%/40 °C, 21.1%/50 °C). Thereby, these low-cost green adsorbents showed high performance in removing RB molecules (as a cationic dye) in alkaline medium, at same time that the pH of 6 also could be concerned as an optimal pH for efficient removal of RB molecules onto these low-cost green adsorbents. Notably, this pH is near that of aqueous medium, which is favorable for practical applications of organic dye removal using these low-cost green adsorbents.

**Figure 7:**
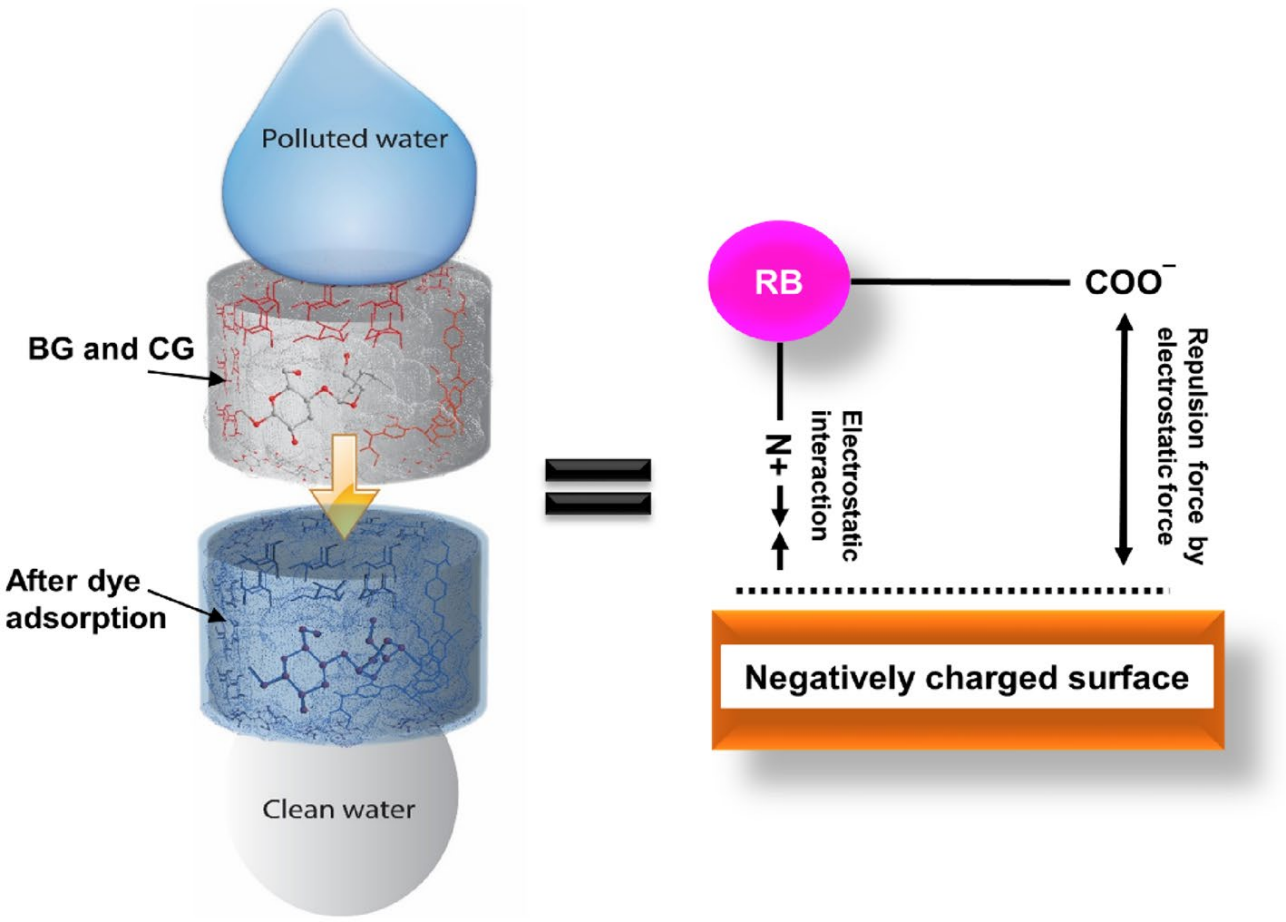
A possible mechanism of RB adsorption onto the non-chemically modified adsorbents.

Here, the RB is dissolved in DI water to release colored dye cations in the solution. The adsorption onto the adsorbent surface from these charged dye groups primarily involves adsorbent surface charges, which is influenced by solution pH^40^, meaning that the utilization of either high or low pH supports to desiredly reach the adsorption efficiency of adsorbate (RB) onto the non-chemically modified adsorbents (BP and CG). Consequently, the positively charged sites (*i.e.,* –OH_2_^+^ and –COOH_2_^+^ groups) existed on the non-chemically modified adsorbents’ surface are not advantageous to support the adsorption of cationic dye molecules at low pH (*i.e.,* the carboxylic and hydroxyl groups of the low-cost green adsorbents were hydrogenated), which can be due to the electrostatic repulsion occurring between RB and the non-chemically modified adsorbents (BP and CG, Fig. 7)^20^. However, the RB removal onto these low-cost green adsorbents still shows high removal ability at low pH. This is because non-electrostatic interactions between RB and the non-chemically modified adsorbents could be stronger than the electrostatic mechanism at low pH, consisting of hydrogen bonds, van der Waals forces, π–π interactions, and hydrophobic–hydrophobic mechanisms^43,44^. Furthermore, –O^−^ and –COO^−^ groups contained on the surface of non-chemically modified adsorbents contribute to the adsorption of cationic dye molecules at high pH^20,45^ (*i.e.,* the carboxylic and hydroxyl groups of the low-cost green adsorbents were dehydrogenated). Otherwise, the RB removal onto these low-cost green adsorbents is low at high pH, which may be regard the inter-molecular interactions, the nature of steric hindrance and the amine group (secondary amine) contained in RB. Such conjunctive pollutant removal will be advantageous for the effluent treatment from various industries because of the well-established chemical nature of pollutants. Concomitantly, the negatively charge carboxyl group is considered as the major functional group in the adsorption of cationic dye molecules, but it is not effective in the adsorption of anionic dye molecules^32,33^.

### Adsorption kinetics

**Figure 8:**
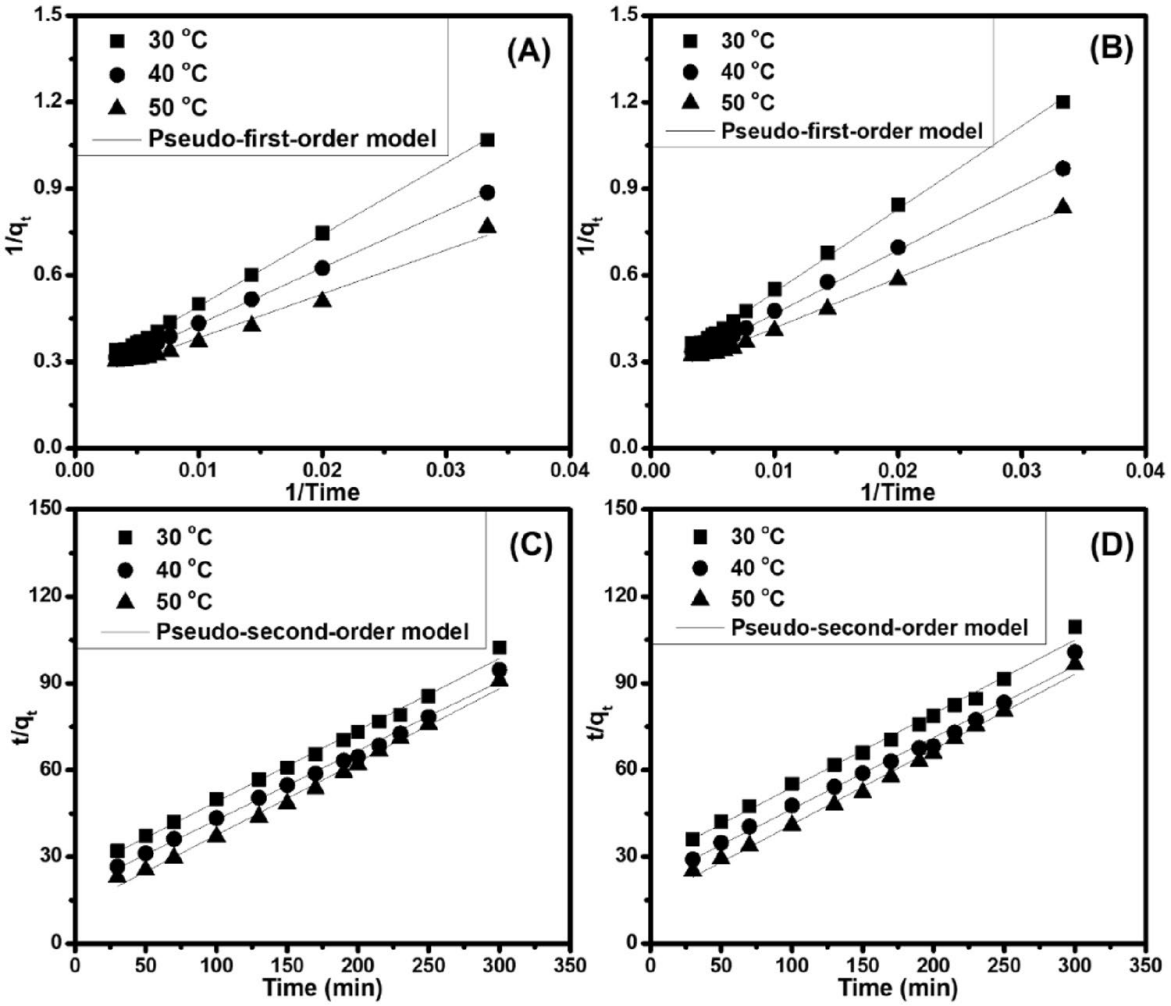
Pseudo-first order (**A, B**) and pseudo-second-order (**C, D**) kinetics for RB adsorption by BP (**A, C**) and CG (**B, D**), respectively.

For wastewater treatment, the investigation of adsorption kinetics and equilibrium is necessary to provide basal information as well as to design and operate the adsorption procedure. Adsorption is known as chemisorption and/or physisorption, regarding a surface phenomenon occurring at the surface or interfacial locations, which involves to mass transfer of a solute from the liquid phase to the adsorbent surface. Typically, the adsorption rate and equilibrium time investigated in the adsorption kinetics are important factors for determining the adsorption mechanism. Here, the adsorption capability of the low-cost green adsorbents was performed at various temperatures with the adsorption time range of 0–300 min according to the RB concentration of 20.0 mg L^−1^, in which two common kinetic models (*i.e.,* pseudo-first-order Eq. (4) and pseudo-second-order Eq. (5)) were applied to investigate the RB adsorption mechanism. More specifically, the widely used rate equation of pseudo-first-order model involves to the sorption of a solute from a liquid solution, while that of pseudo-second-order model is based on sorption equilibrium capacity. As shown in Table 1 and Fig. 8, the pseudo-second-order equation is a well fitted one to compare the experimental data of the whole adsorption period at different temperatures, and its regression coefficient (R_2_) is closer to 0.999 for the concentration range used in this study. Hence, the RB adsorption kinetics occurring onto the non-chemically modified adsorbents (BP and CG) is in accordance with the pseudo-second-order model, indicating that the rate-limiting step could be regarded to chemical adsorption. Therefore, the RB adsorption involves surface exchange reactions until the fully occupied surface functional sites, and then RB molecules will diffuse into the network of the adsorbent (BP and CG) to continue the interaction.

**Table 1:** Adsorption kinetics and isotherm parameters for RB adsorption onto the non-chemically modified adsorbents.

Sample	Temp. (°C)	q_e,exp_ (mg g^−1^)	Pseudo-first-order model			Pseudo-second-order model		
			q_e,cal_ (mg g^−1^)	k_1_ (min^−1^)	R^2^	q_e, cal_ (mg g^−1^)	k_2_ (× 10^−4^ g mg^−1^ min^−1^)	R^2^
BP	30	4.30 ± 0.21	4.10	101.93	0.995	4.04	25.31	0.999
	40	4.59 ± 0.25	4.27	83.75	0.993	4.18	30.47	0.999
	50	5.04 ± 0.34	4.34	66.20	0.984	3.95	52.49	0.993
CG	30	4.06 ± 0.25	3.95	113.78	0.992	3.93	22.77	0.998
	40	4.41 ± 0.42	4.07	89.88	0.990	4.04	28.14	0.997
	50	4.83 ± 0.30	4.11	71.52	0.994	3.84	44.85	0.995

Sample	Temp. (°C)	q_e, exp_ (mg g^−1^)	Langmuir model			Freundlich model		
			q_m, cal_ (mg g^−1^)	K_L_ (L mg^−1^)	R^2^	n	K_F_ (L mg^−1^)	R^2^
BP	30	4.30 ± 0.21	6.76	0.17	0.980	3.39	1.76	0.855
	40	4.59 ± 0.25	6.96	0.30	0.994	3.84	2.28	0.792
	50	5.04 ± 0.34	7.64	0.43	0.996	4.11	2.81	0.842
CG	30	4.06 ± 0.25	6.53	0.13	0.964	3.24	1.53	0.871
	40	4.41 ± 0.42	6.80	0.23	0.994	3.31	1.83	0.846
	50	4.83 ± 0.30	7.51	0.27	0.995	3.27	2.06	0.871

In addition, in the pseudo-second-order model, rate constants (k_2_, Table 1) have been used to determine the activation energy (E_a_, J mol^−1^) of RB adsorption on the non-chemically modified adsorbents (BP and CG) by using the Arrhenius equation [Eq. (6)], and its linear form is shown in Eq. (7^21,46^: R (8.314 J mol^−1^ K^−1^), k_o_ (g mg^−1^ min^−1^), and T (K) indicate the gas constant, temperature-independent factor, and tested temperature, respectively.

4 $$\frac{1}{{\mathrm{q}}_{\mathrm{t}}}=\frac{{\mathrm{k}}_{1}}{{\mathrm{q}}_{\mathrm{e}}\mathrm{t}}+\frac{1}{{\mathrm{q}}_{\mathrm{e}}}$$

5 $$\frac{\mathrm{t}}{{\mathrm{q}}_{\mathrm{t}}}= \frac{1}{{\mathrm{k}}_{2}{\mathrm{q}}_{\mathrm{e}}^{2}}+ \frac{\mathrm{t}}{{\mathrm{q}}_{\mathrm{e}}}$$

6 $${\mathrm{k}}_{2}= {\mathrm{k}}_{\mathrm{o}}{\mathrm{e}}^{\frac{{-\mathrm{E}}_{\mathrm{a}}}{\mathrm{RT}}}$$

7 $${\mathrm{lnk}}_{2}= \frac{{-\mathrm{E}}_{\mathrm{a}}}{\mathrm{RT}}+\mathrm{ ln}{\mathrm{k}}_{\mathrm{o}}$$

The adsorption system has two major types: physical adsorption and chemical adsorption (activated and non-activated ones). More particularly, the activated chemical adsorption indicates that the rate changes with different temperatures in accordance with E_a_ in the Arrhenius equation, in which this equation is used to associate the rate constant and the kinetic model’s activation energy at various temperatures. By contrast, E_a_ in non-activated chemical adsorption is near zero. Usually, the types of interactions between RB and the low-cost green adsorbents relate to the physical or chemical adsorption process, in which the magnitude of E_a_ shows whether the adsorption is mostly chemical or physical. Based on Eq. (7), E_a_ has positive values of 29.51 kJ mol^−1^ (BP) and 27.46 kJ mol^−1^ (CG) based on the determined slope from the Arrhenius plot of lnk_2_ *versus* 1/T. Therefore, increasing the temperature will promote adsorption, as well as this process, which is considered as an endothermic one (E_a_ > 0). In addition, the major types of adsorption could be based on the calculated activation energy value. The activation energy range of 5–40 kJ mol^−1^ will reveal physical adsorption, thus, the process interaction in such a system is readily reversible and equilibrium is rapidly established, which are because of the modest intermolecular interactions. In the meantime, the activation energy range of of 40–80 kJ mol^−1^ will indicate chemical adsorption ^47,48^, meaning that chemisorption experiences stronger bonding forces. In this study, the adsorption activation energy for RB has confirmed the physical adsorption of RB on the surface of non-chemically modified adsorbents [*i.e.,* 29.51 kJ mol^−1^ (BP) and 27.46 kJ mol^−1^ (CG)], showing that the adsorption has a low potential barrier. The results indicate that RB adsorption has occurred during chemical and physical adsorptions through the pseudo-second-order model and the activation energy range of of 40–80 kJ mol^−1^, respectively.

### Adsorption isotherms

**Figure 9:**
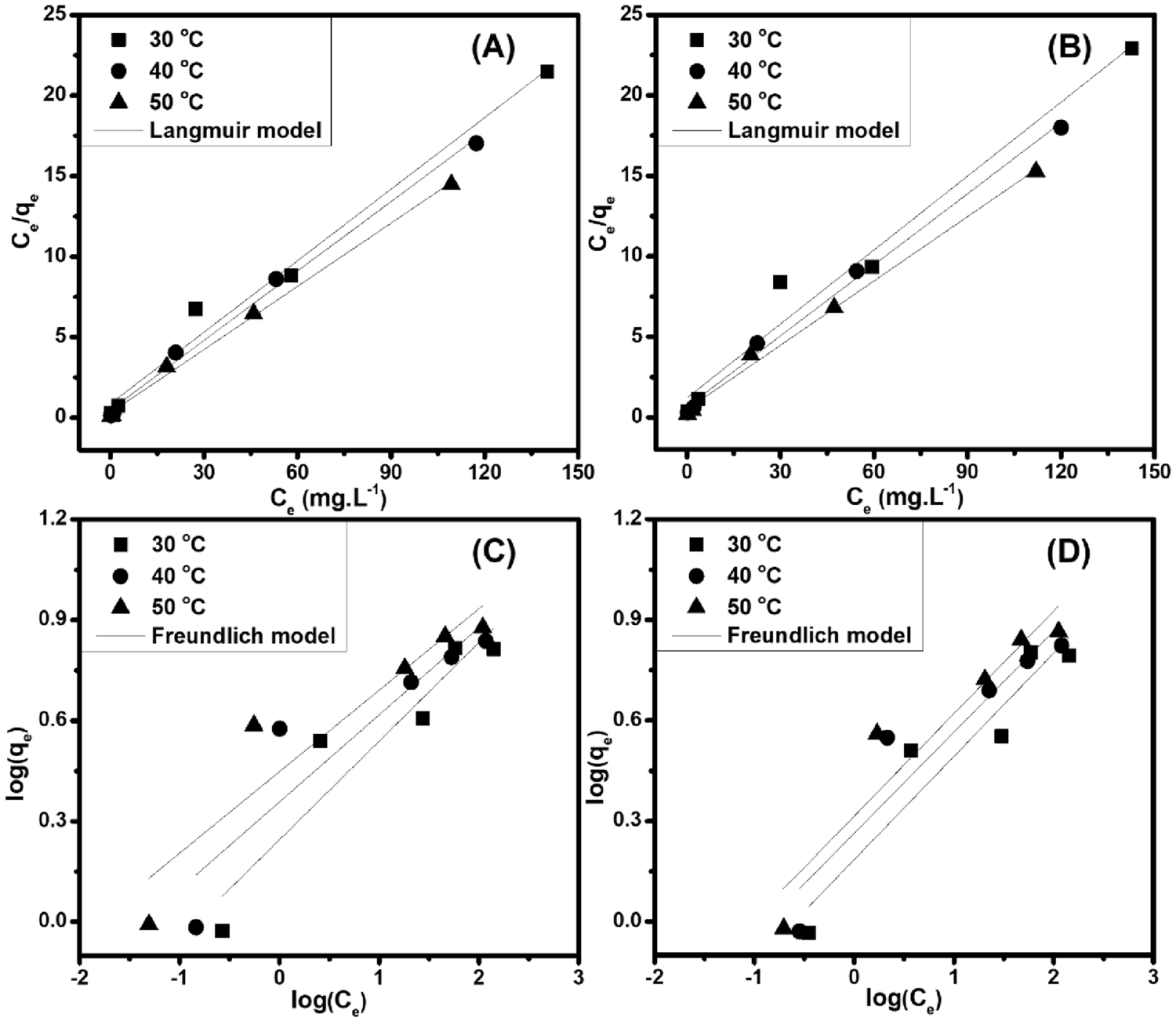
Langmuir (**A, B**) and Freundlich (**C, D**) isotherms for RB adsorption by BP (**A, C**) and CG (**B, D**), respectively.

The design of the RB adsorption system can constitute the most optimal correlation for the equilibrium curves, in which adsorption isotherms are used to describe the possible relationship between the adsorbed amounts of adsorbate on the surface of adsorbent. Here, the adsorption capability of the low-cost green adsorbents was determined at various temperatures during the adsorption time of 230 ​min according to the different RB concentrations (5–200 mg L^−1^). Regarding the adsorption isotherms, two widely used models, *i.e.,* Langmuir [Eq. (8)] and Freundlich [Eq. (9)] equations, for determining the mechanistic parameters relating to the RB adsorption process onto the non-chemically modified adsorbents at the above-mentioned various temperatures. Among them, Langmuir model is based on the adsorbate’s mono-layer coverage of on the adsorbent’s homogeneous surface, while Freundlich model relates to the multi-layer adsorption on the adsorbent’s heterogeneous surface. As shown in Table 1 and Fig. 9, the Langmuir isotherm is well fitted (R_2_ ~ 0.99) in the entire adsorption period at different temperatures, thereby indicating the homogeneous nature of the surface of non-chemically modified adsorbents. Furthermore, the increase in the tested temperature has improved the adsorption capacity (*i.e.,* BP: 6.76 mg g^−1^/30 °C, 6.96 mg g^−1^/40 °C, 7.64 mg g^−1^/50 °C and CG: 6.53 mg g^−1^/30 °C, 6.80 mg g^−1^/40 °C, 7.51 mg g^−1^/50 °C).

Obviously, the adsorption capacity increases with temperature. Hence, the Gibbs-free energy (ΔG°, kJ mol^−1^), entropy (ΔS°, kJ mol^−1^ K^−1^), and enthalpy (ΔH°, kJ mol^−1^) values are determined at the above-mentioned temperatures based on the van’t Hoff equation [Eq. (10)]; R (8.314 J mol^−1^ K^−1^), K_L_ (m^3^ mol^−1^), and T (K) refer to a gas constant, Langmuir constant, and tested temperature, respectively. In particular, ΔH° and ΔS° values are based on a determined slope of the linear plot of ΔG° *versus* T.

8 $$\frac{{\mathrm{C}}_{\mathrm{e}}}{{\mathrm{q}}_{\mathrm{e}}}=\frac{1}{{\mathrm{q}}_{\mathrm{m}}{\mathrm{K}}_{\mathrm{L}}}+\frac{{\mathrm{ C}}_{\mathrm{e}}}{{\mathrm{q}}_{\mathrm{m}}}$$

9 $${\mathrm{logq}}_{\mathrm{e}}={\mathrm{logK}}_{\mathrm{F}}+\frac{1}{\mathrm{n}}{\mathrm{logC}}_{\mathrm{e}}$$

10 $${\Delta \mathrm{G}}^{\mathrm{o}}= -{\mathrm{RTlnK}}_{\mathrm{L}}={\Delta \mathrm{H}}^{\mathrm{o}}-{\mathrm{T}\Delta \mathrm{S}}^{\mathrm{o}}$$

According to theorical thermodynamics, Gibbs energy (G) is known as possible changes regarding the Helmholtz free energy, which usually occur in a closed system at fixed temperature and pressure. The Gibbs energy can be employed to reveal whether changes happen spontaneously or if they are forced. More obviously, there will be three important cases regarding changes in Gibbs energy, such as (i) the changes in closed systems are spontaneous (ΔG < 0), (ii) the changes are reversible or the systems are in equilibrium (ΔG = 0), and (iii) the changes are forced (ΔG > 0). As summarized in Table 2, the negatively attained ΔG° values show the spontaneous nature of RB adsorption at different temperatures, the affinity between the non-chemically modified adsorbents (BP and CG), and the adsorbate (RB). In addition, the positive values of ΔH° (BP 37.44 kJ mol^−1^ and CG 30.77 kJ mol^−1^) and ΔS° (BP 0.1604 kJ mol^−1^ K^−1^ and CG 0.1362 kJ mol^−1^ K^−1^) indicate the endothermic properties of adsorption and the increased randomness at the interface between the solid–liquid phases, respectively. Moreover, the attained ΔH° values are less than 40 kJ mol^−1^, which indicates physical adsorption^48^, but those in the range of 40–800 kJ mol^−1^ indicate chemical adsorption^47,48^. Thus, these results are similar to the above-mentioned RB adsorption that occurs during physical and chemical adsorptions. For various adsorbents, based on the adsorption isotherms, the saturated adsorption capacity for adsorbate could be compared by determining the amount of adsorbate adsorbed on the adsorbents. As listed in Table 3, the adsorption capacity of non-chemically modified adsorbents in this study is greater than that of modified waste materials (trichoderma harzianum mycelial waste [THMW], cellulosic waste orange peel [CWOP], and coal ash [CA]) and modified natural materials (Australian natural zeolite [ANZ], zeolite from fly ash-iron oxide magnetic nanocomposite [ZM], and Na^+^-montmorillonite [Na^+^-MMT])^44,49–53^. Therefore, the non-chemically modified materials or the reuse of CG are potential candidates that are directly applied as low-cost adsorbents in wastewater treatment.

**Table 2:** Thermodynamic parameters of RB adsorption onto the non-chemically modified adsorbents.

Sample	Temp. (°C)	K_L_ (m^3^ mol^−1^)	∆G° (kJ mol^−1^)	∆H° (kJ mol^−1^)	∆S° (kJ mol^−1^ K^−1^)
BP	30	81.64	− 11.090	37.44	0.1604
	40	141.71	− 12.891		
	50	205.23	− 14.298		
CG	30	60.67	− 10.342	30.77	0.1362
	40	108.02	− 12.185		
	50	129.76	− 13.066

**Table 3:** Dye adsorption capacity of various adsorbents.

Adsorbent	Adsorbate	Contact time (min)	Adsorbent dose (g L^−1^)	Temp. (°C)	pH	Isotherms	Kinetics	Thermodynamics	Adsorption capacity (mg g^−1^)	References
Non-chemically modified BP	RB	230	6	30, 40, 50	6	Langmuir	Pseudo-second order	Spontaneous endothermic	6.76, 6.96, 7.64	This work
Non-chemically modified CG	RB	230	6	30, 40, 50	6	Langmuir	Pseudo-second order	Spontaneous endothermic	6.53, 6.80, 7.51	This work
THMW	RG	120	8	30		Langmuir	Pseudo-second order	–	3.40	^49^
CWOP	RB	45	10	29 ± 2	5.2	Langmuir, Freundlich	Pseudo-first order	–	3.23	^50^
	PO	45	10	29 ± 2	5.2	Langmuir, Freundlich	Pseudo-first order	–	1.33	^50^
CA	RB	–	–	30	6.2	Langmuir, Freundlich	–	–	2.86	^51^
	MB	–	–	30	6.2	Langmuir, Freundlich	–	–	2.26	^51^
ANZ	RB	–	0.25	30, 50	6	Langmuir, Freundlich	Pseudo-second order	Spontaneous endothermic	2.10, 2.76	^44^
	MB	–	0.25	30, 50	6	Langmuir, Freundlich	Pseudo-second order	Spontaneous endothermic	6.80, 7.91	^44^
ZM	RO	60	0.05	25–40	–	Langmuir	Pseudo-second order	Spontaneous endothermic	1.06	^52^
	IC	60	1	25–40	–	Langmuir	Pseudo-second order	Spontaneous endothermic	0.58	^52^
Na^+^-MMT	RG	10	5	18–34	6	Freundlich, Dubinin-Radushkevich	Pseudo-first order	Exothermic	0.40	^53^

THMW: Trichoderma harzianum mycelial waste, CWOP: cellulosic waste orange peel, CA: coal ash, ANZ: Australian natural zeolite, ZM: zeolite from fly ash-iron oxide magnetic nanocomposite, Na^+^-MMT: Na^+^-montmorillonite, RG: rhodamine 6G, PO: procion orange, MB: methylene blue, RO: reactive orange 16, IC: indigo—carmine.

### Characterization of the used non-chemically modified adsorbents

**Figure 10:**
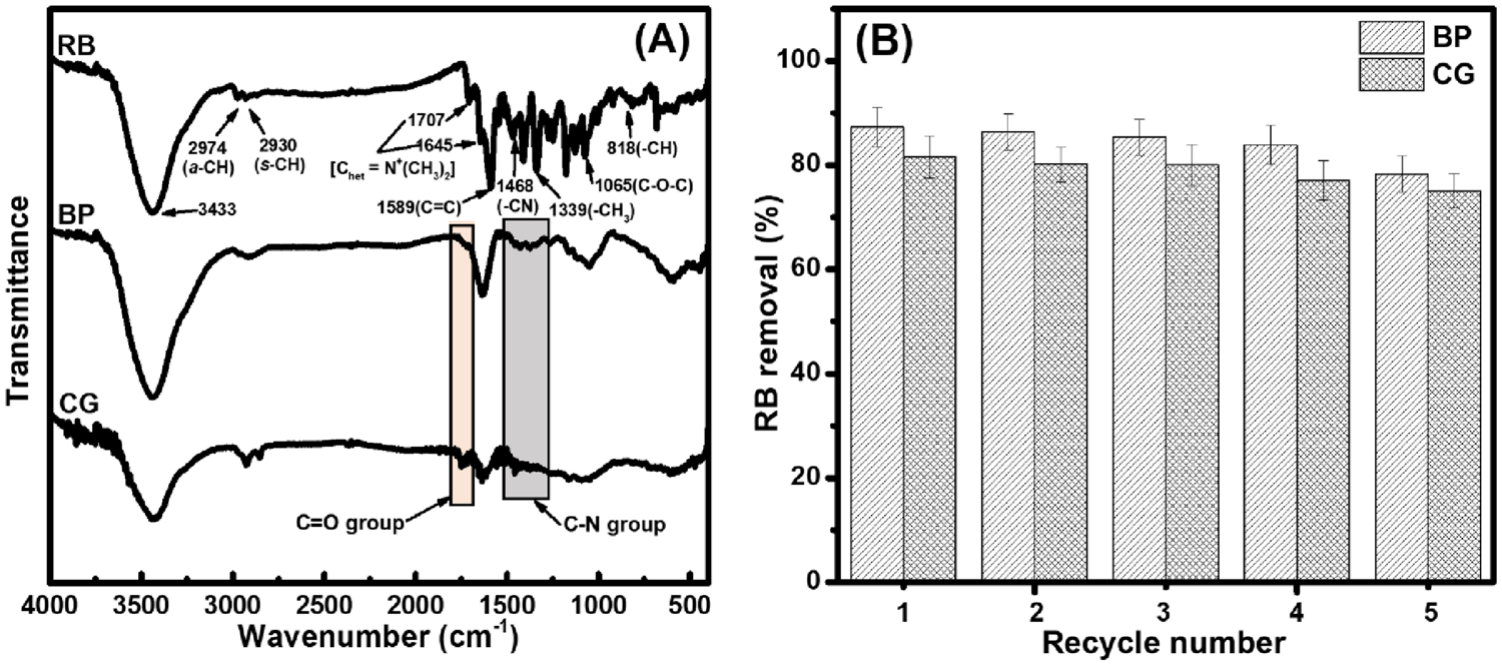
FTIR spectra of pure RB and RB-loaded adsorbents (**A**). Repeated RB removal efficiency of the non-chemically modified adsorbents during recycling (**B**).

In addition, the RB adsorption mechanism can be based on the FTIR spectra of pure RB and RB-loaded adsorbents (Fig. 10A). In particular, in pure RB spectra, a broad peak at 3433 cm^−1^ belongs to the –OH/–NH stretching vibration. The peaks at 2974 and 2930 cm^−1^ indicate asymmetric and symmetric –CH stretching vibrations, respectively. Two weak peaks at 1645 and 1707 cm^−1^ are related to C_het_ = N^+^(CH_3_)_2_ stretching vibrations; concomitantly, a peak at 1589 cm^−1^ indicates C=C vibration in the aromatic ring structure^54^. Moreover, the peaks at 1468, 1339, and 1065 cm^−1^ are attributed to C–N stretching [–*CN*(C_2_H_5_)_2_], –CH_3_ bending, and C–O–C stretching (an aromatic ring) vibrations, respectively ^55,56^. In particular, a characteristic peak of–C–H blending vibration appearing in the di-substituted benzene ring of the RB molecule was also observed at 818 cm^−1^^57^. By contrast, the FTIR spectra of RB-loaded adsorbents have few changes in characteristic peaks, which might reveal relatively weak forces during RB adsorption. Notably, the –OH/–NH stretching vibrations are broader, and they shift to low wavenumber regions after RB adsorption (*i.e.,* 3423 to 3414 cm^−1^ [BP] and 3441 to 3429 cm^−1^ CG]), which is related to the potential hydrogen bonds between the non-chemically modified adsorbents (hydroxyl groups) and RB. Concomitantly, the C=O stretching vibration of non-chemically modified adsorbents (1740–1605 cm^−1^ [BP] and 1744–1628 cm^−1^ [CG]) and C–N stretching vibrations of RB (1468 cm^−1^) are attenuated, which show charge neutralization between the –COO^−^ ions of adsorbents and –N^+^ ions of RB^58^. Therefore, possible interactions are intervened among the functional groups of non-chemically modified absorbents (BP and CG) and the adsorbate (RB), thereby supporting the above-mentioned isotherm data.

### Recycling performance

In addition to cost-effective utilization of the non-chemical-modified green absorbents (BP and CG), recycling performance of an adsorbent is also considered as an important requirement in practical applications, regarding the ecological and economic needs for its sustainability. It means that a low-cost green adsorbent not only possesses a high dye removal performance, but also shows good desorption ability to lower the total cost. In upholding the adsorption capacity during their repeated utilization in wastewater treatment, the reuse performance of non-chemically modified absorbents (BP and CG) was investigated at 30 °C during the reuse process, which is conducted by immersing and washing the RB-loaded BP and CG into ethanol solvent to remove RB molecules after each adsorption process. Then, these samples were used to re-adsorb the RB (20 mg L^−1^) at 230 min. In several recent correlative works, CWOP, which is a modified waste material ^50^, has shown good adsorption ability in an acidic medium (acetic acid) and good desorption ability in an alkaline medium (pH 12.0) for anionic dyes (CR and procion orange). Thus, these anionic dyes were held by the CWOP (*i.e.,* ion exchange). However, for cationic dye (RB), the desorption performance did not remarkably change (17.0–27.0%) with the increase of pH from 3.0 to 11.0, as well as it is similar to the adsorption ability, indicating that ion exchange could not be considered as a major part in the adsorption process. In addition, THMW, which is a modified waste material^49^ obtained from 0.1 to 0.5 N NaOH, successfully desorbed rhodamine 6G (*i.e.,* 26.9–57.9%). Therefore, THMW can be regenerated and recycled. In particular, the non-chemically modified BP and CG as low-cost adsorbents have not been identified for recycling performance.

Here, the RB removal percentage of non-chemically modified adsorbents almost did not decrease in each RB solution from the 1st cycle to the 5th cycle; however, their RB removal percentage slightly decreases from the 4^th^ cycle to the 5th cycle (*i.e.,* 83.8–78.3% [BP] and 80.0–75.0% [CG], Fig. 10B), regarding a decrease in recyclability of the low-cost green adsorbents. It means that a decrease in the functional groups and stability of these adsorbents’ active sites in the whole of their structure. Therefore, these low-cost green adsorbents are considered as reusable adsorbents in five cycles. As such, BP and CG are used as low-cost materials (particularly as zero-cost adsorbent) and suggested as potential adsorbents because of the following characteristics: (i) high recycling ability, (ii) easy recycling stage, and (iii) nearly zero cost of their preparation (the non-chemically modified form)^59^. Hence, the above-mentioned points could provide a cost potential for their applications in water/wastewater treatment.

## Conclusions

The natural abundance materials such as BP and CG are used directly without the chemical modification for RB adsorption. The adsorption capacity of RB using low-cost adsorbents (non-chemically modified BP and CG) is evaluated during adsorption. Several effects of RB adsorption conditions onto the low-cost green adsorbents were investigated, such as contact times, temperatures, doses of the non-chemically modified adsorbents, initial RB concentrations, and pH values of RB solution. In addition, the experimental data is well fitted acoording to the pseudo-second-order model. The positive values of E_a_ [*i.e.,* 29.51 kJ mol^−1^ (BP) and 27.46 kJ mol^−1^ (CG)] indicate that the increase in temperature supports adsorption, and the adsorption process involves endothermic properties. Furthermore, the Langmuir model is well appropriately with the experimental data for all samples; the maximum adsorption capacities are 6.76, 6.96, and 7.64 mgg^−1^ for BP, as well as 6.53, 6.80, and 7.51 mgg^−1^ for CG at 30 °C, 40 °C, and 50 °C, respectively. The attained ΔG° with negative values (*i.e.,* from − 11.09 to − 14.30 kJ mol^−1^ [BP] and from − 10.34 to − 13.07 kJ mol^−1^ [CG]) reveal the spontaneous nature of the adsorption phenomena; concomitantly, the values of ΔH° and ΔS° are calculated to be 37.44 kJ mol^−1^/0.1604 kJ mol^−1^ K^−1^ (BP) and 30.77 kJ mol^−1^/0.1362 kJ mol^−1^ K^−1^ (CG) correspondingly. Therefore, the RB adsorption is an endothermic reaction, which is at the state of physical and chemical adsorptions, and recycling ability of these low-cost green adsorbents is determined in five cycles. As such, these probably provide a cost potential of these non-chemically modified BP and CG for applications in water/wastewater treatment.

## Data Availability

The data obtained and/or analyzed during the current study are available from the corresponding author on reasonable request.
